# Development and validation of a screening instrument to assess the types and quality of foods served at home meals

**DOI:** 10.1186/1479-5868-9-10

**Published:** 2012-02-07

**Authors:** Jayne A Fulkerson, Leslie Lytle, Mary Story, Stacey Moe, Anne Samuelson, Audrey Weymiller

**Affiliations:** 1 School of Nursing, University of Minnesota, 5-160 Weaver-Densford Hall, 308 Harvard Street SE, Minneapolis, MN 55455, USA; 2 Division of Epidemiology & Community Health, University of Minnesota, 1300 South Second Street, Suite 300, Minneapolis, MN 55454, USA; 3 Human Development & Family Studies, University of Wisconsin-Madison, Madison, USA

**Keywords:** Validation, Meal screener, Dinner, Home, Families, Food

## Abstract

**Background:**

Although there is growing interest in assessing the home food environment, no easy-to-use, low cost tools exist to assess the foods served at home meals, making it difficult to assess the meal component of the food environment. The aim of this study was to develop and validate a user-friendly screener to assess the types of foods served at home meals.

**Methods:**

Primary food preparing adults (n = 51) participated in a validation study in their own homes. Staff and participants independently completed a screener as participants cooked dinner. The screener assessed the types of foods offered, method(s) of preparation, and use of added fats. Two scale scores were created: 1) to assess offerings of foods in five food groups (meat and other protein, milk, vegetables, fruit, grains), 2) to assess the relative healthfulness of foods based on types offered, preparation method, and added fats. Criterion validity was assessed comparing staff and participant reports of individual foods (kappa (k)) and scale scores (Spearman correlations).

**Results:**

Criterion validity was high between participants' and staffs' record of whether major food categories (meat and other protein, bread and cereal, salad, vegetables, fruits, dessert) were served (k = 0.79-1.0), moderate for reports of other starches (e.g., rice) being served (k = 0.52), and high for the Five Food Group and Healthfulness scale scores (r = 0.75-0.85, *p *< .001).

**Conclusions:**

This new meal screening tool has high validity and can be used to assess the types of foods served at home meals allowing a more comprehensive assessment of the home food environment.

## Background

Studies have shown that compared to foods consumed at home, away-from-home foods are higher in fat and calories [1] and contribute to poorer dietary quality and overweight status [2-6]. Thus, health and nutrition experts recommend limiting eating out and encourage more frequent home meal preparation [7]. The importance of the home environment in influencing food intake and weight status has prompted the development of new valid instruments to assess food availability within the home food environment [8,9]. Although these instruments assess foods available in the home, no validated instruments exist to assess what types of foods are served specifically at meals within the home. Foods served at meals may include a subset of those available within the home. Moreover, little is known about how these foods are prepared which may be important in regard to fat content.

The family meal component of the home food environment has been gaining national attention, particularly because research has shown that family meals positively impact the dietary intake of children [10-17] and may be associated with overweight status, particularly among young children [10,15,18,19]. Because almost 70% of calories and 80% of snacks consumed by children ages 6-11 years are eaten in the home [2], developing measures to assess the types of foods served at home meals is an important first step in gaining a better understanding of the influence of the home environment on children's food intake [20]. Although assessment of dietary intake at specific meals could be conducted with traditional methods of dietary recall interviews, this methodology is expensive and time- and labor-intensive. Thus, a practical, easy-to-use valid instrument is needed for these assessments as the health promotion field encourages and advocates healthy lifestyle changes for families.

The purpose of this study was to develop and validate a self-administered screening instrument to assess the types of foods served at meals in the home setting. Additional goals included developing an instrument that was easy to self-administer using a format that captured a variety of foods, and that could provide summary indicators of food quality.

## Methods

### Procedures

Primary meal preparing adults (one per home where a child between the ages of 8 and 18 years resided) were recruited from the community using flyers posted at 19 Minneapolis Park and Recreation Centers to complete the screening instrument as they made a typical evening meal ("meal" was undefined) in their home. Participants were also invited to participate in three other studies at the time of screening, including validation of a home food inventory [9] and validation of home physical activity and media equipment [21] using similar methodology. Trained research staff traveled to the participants' homes to obtain written consent and independently complete the instrument while observing the participant preparing the meal. The screener typically took 5-15 min to complete, depending on the number of ingredients included in the meal. Participants did not receive "training" on how to complete the screener as the intent was for the screener's written directions to be self-explanatory to facilitate independent completion. Instructions indicated to list all foods and beverages prepared or made available as part of the evening meal, even if only one person ate it. Participants received a $30 gift card for their participation. The University of Minnesota's Institutional Review Board approved this study.

### Participants

The validation sample consisted of 51 adults aged 23-53 years (M = 39.4, SD = 7.0; 94% female). Sixty-eight percent of the sample was white, followed by African American (14%), American Indian (6%), mixed race/ethnicity (6%), Latino (4%), and Asian (2%). Over half (62%) had a college degree, 26% had some college or vocational training and 12% had a high school degree or less.

### Measures

#### Meal screener: development

Development of the screening instrument began with the investigators drafting items to reflect categories of foods that might be served at home meals and those likely to be useful to assess relative healthfulness. Preset food categories were used to facilitate instrument completion, scoring and analysis. Opinions from four internationally-respected nutrition experts were requested for further instrument development and assessment of face validity (see acknowledgements). The instrument was then revised to more finely discriminate between more and less healthful foods (e.g., by providing more options for sauces that were clearer in regard to fat content), account for mixed dishes, and clarify the instructions. Field testing of the instrument was conducted with five adults to inform revisions for the final version regarding ease of completion and to identify any foods that were difficult to include on the form.

#### Meal screener: final version

The final screener included an open-ended section for participants to write in foods that were served at the evening meal. Examples were provided to indicate that they should list main course, side dishes, beverages and dessert, if applicable. This initial step provided the participant with a reminder of what was served to assist with subsequent questions regarding preparation. The next section of the screener asked specific questions about the types of foods served and method of preparation in preset major food categories: 1) meat or other protein, 2) bread or cereal, 3) starches other than bread (e.g., pasta, noodles, potatoes, rice, pizza dough), 4) salad, 5) vegetables (other than potato), 6) fruit, 7) dessert, and 8) beverages. Foods within each major food category (e.g., pork (as food subcategory) within the meat/protein major food category) were presented in a checklist format (yes/no if served) and included a checklist for preparation options and added fats (e.g., butter, sauce).

Table 1 describes the specific foods included in each major food category. For example, for the protein category, a participant would check "yes" if meat or other protein was served (i.e., served major food category) and then be prompted to check the specific type of food that was served within that category (e.g., poultry food subcategory). Then, he/she was instructed to check response options regarding method of preparation and whether or not fats were added during the cooking or serving process.

**Table 1:** Description of major food categories, food subcategories, method of preparation and added condiments, sauces, fats^a^

Major food categories and food subcategories	Method of preparation	Added condiments, sauces and fats
**Meat or other protein**		
Poultry Beef Pork/ham Fish/shellfish Lamb/veal Veggie burgers Tofu, seitan, tempe, TVP or other soy Lentils, beans Peanut butter or other nut butter Egg or egg substitute Other meat or protein	Not cooked/raw Boiled/steamed Grilled Roasted/baked/broiled Sautéed/fried Deep fried other	Cream or oil based* Gravy* Tartar sauce* Steak or other meat sauce Tomato-based sauce Ketchup or other condiment Broth/stock Salsa Other

**Bread and cereal**		
Garlic bread or other bread with cheese or cheese sauce White bread or rolls Flour tortillas Corn tortillas Pita bread Biscuits or croissants Whole grain cereal Sugared cereal Low sugar cereal Other		Regular butter or margarine* Reduced-fat or light butter or margarine Other sauce

**Other starches**		
Pasta/noodles Potato Rice Pizza dough Other	Not cooked/raw Boiled/steamed Baked/roasted Fried Other	Butter or margarine* Cheese or other sauce Cream or oil based* Cheese based* Tomato sauce (meat)* Tomato sauce (no meat) Ketchup or condiment Other

**Salad**		
Salad greens Avocado Beets Bell peppers Broccoli Cabbage Carrots Cauliflower Celery Cheese Cucumbers Green beans Jicama Oranges Mushrooms Onions Pears Peas Raisins Spinach or other greens Tomatoes Other		Regular salad dressing* Low-fat, light or non-fat salad dressing Oil and vinegar* Other

**Vegetables**		
Mixed vegetables Asparagus Beets Bell peppers Broccoli Cabbage Carrots Cauliflower Celery Corn	Not cooked/raw Boiled/steamed/microwaved Baked/roasted/broiled/grilled Fried/sautéed Other	Cheese sauce* Oil* Condensed soup or similar sauce* Regular salad dressing or dip* Low-fat, light or nonfat salad dressing or dip Other
Cucumbers Green beans Jicama Mushrooms Onions Peas Spinach or other greens Squash Tomatoes Other		

**Fruits**		
Apples Apple sauce Apricots Avocado Bananas Berries Dates/figs Grapes Grapefruit Kiwi Mango Melon Mixed fruit/fruit cocktail Nectarines Oranges or other citrus Pears Peaches Pineapple Plums Prunes/raisins Other	Not cooked/raw Baked/roasted/broiled/grilled Other	Cream based sauce* Regular chocolate or caramel sauce* Light, low-fat or non-fat whipped cream, chocolate or caramel Sugar Other

**Beverages**		
Milk (whole/2%) Milk (1%/fat free/skim) Chocolate milk		
Reduced-fat yogurt drinks Soy or other nondairy milk Water Sweetened water 100% fruit juice Juice blend Sports drinks Soda pop (regular) Soda pop (diet) Tea/coffee Other		

**Dessert**		
Cookies Cake/cupcakes Brownies/bars Ice cream Pudding Pastry/doughnuts Candy/chocolate Yogurt Fruit-based dessert Fresh fruit Other		Cream based sauce* Regular chocolate or caramel* Light, low-fat, non-fat whipped cream, chocolate or caramel Other

^a ^instructions and examples not provided

*calculated as an added fat in scoring

#### Scale scores

Two scale scores were created to summarize home meal quality; one to assess offerings of foods within the five major food groups of the Food Guide Pyramid [22] (meat or other protein, milk, vegetables, fruit, grains) and another to assess the healthfulness of foods based on types of foods offered, preparation method, and added fats. For the Five Food Group score, participants were given one point for serving at least one food in each food group (range = 0-5). To more fully examine food offerings to include methods of food preparation and added fats, for the Healthfulness scale score, participants were given a point for serving a food from each of the major food categories and a point for a healthy preparation method (e.g., baking); a point was subtracted if a high-calorie sauce was added (range = 0-10). The screener is available from the corresponding author upon request.

### Data analysis

Criterion validity was assessed by comparing participants' and research staffs' responses on the screener. Consistent in research of criterion validity, the research staff report was considered the "gold standard" [23] as they were trained on how to use the screener. Kappa and Spearman correlation statistics were used to evaluate these comparisons for individual foods, food categories and scale scores (Five Food Group and Healthfulness scores), respectively. Kappa statistics greater than 0.60 reflect substantial agreement. To summarize these results, we calculated the average kappas (across individual foods) within major food categories. All analyses were conducted in SAS (v9.1, SAS Institute, Inc., Cary, NC, 2003).

## Results

As shown in Table 2, the most frequently served major food categories at the home evening meal were meat or other protein and vegetables. Less than half of participants reported serving bread, salad or fruits. Dessert was served by about half of participants while beverages were served by almost all participants. In regard to the Five Food Group score, about one-third (37%) of participants reported serving foods from four of the five food groups, followed by foods from three food groups (27%), five food groups (18%), and two food groups (12%); 4% reported serving from one food group, and 2% did not serve foods from any of the five food groups. The Healthfulness scale score average was about 5 out of 10 (M = 4.6, SD = 1.8).

**Table 2:** Prevalence of serving foods in major food categories and criterion validity of major food categories, food subcategories, method of preparation and use of added fats (n = 51)

	Participant reported served	Agreement (kappa) between staff and participant			
**Food category**		**Major food category served/not served^a^**	**Food subcategories served/not served^b^**	**Preparation method^c^**	**Added fats^d^**

Meat or other protein	96%	1.0	0.87	0.76	0.58

Beverages	96%	--	0.84	N/A	N/A

Vegetables^e^	82%	0.79	0.84	0.53	0.81

Other starch^f^	55%	0.52	0.76	0.76	0.59

Dessert	53%	0.88	0.85	N/A	--

Bread	47%	0.79	0.75	N/A	0.82

Salad	39%	0.96	0.84	N/A	--

Fruits	35%	0.81	0.74	0.77	--

^a ^kappa comparing staff and participant report of whether major food category was served

^b ^kappa comparing staff and participant report of whether subcategory foods were served (averaged across foods within same subcategory)

^c ^includes raw/not cooked, boiled/steamed, grilled, roasted/baked/broiled, sautéed/fried, deep fried

^d ^includes sauces/condiments for meat or other protein, vegetables and other starches, butter/margarine/other sauce for breads and vegetables, salad dressings for salads, and sauces for fruit and dessert

^e ^includes vegetables other than potato

^f ^includes pasta/noodles, potato, rice, pizza dough, and other

^--^unable to calculate kappa because all participants reported that beverages were always served; all staff reported all salads were served with dressing; no one reported fruits or desserts served with sauce

N/A = Not applicable (preparation methods and/or added fats were not assessed)

Table 2 provides a description of criterion validity (kappa statistics) with comparisons of agreement between the trained staff data (gold standard) and participants' data regarding whether or not a food was served from a major food category (e.g., meat or other protein; column 3), across foods within each major food category (food subcategory, column 4), method of preparation (column 5), and added fats (column 6). Kappa statistics between participants' and staffs' record of whether meat or other protein, beverages, vegetables, dessert, bread, salad, fruits were served ranged from 0.79 (vegetables) to 1.0 (meat or other protein), while the kappa value for serving other starches was 0.52. Average kappa values for each major food category ranged from 0.74 (fruits) to 0.87 (meat or other protein). Average kappa values for method of preparation ranged from 0.53 (vegetables) to 0.77 (fruits) and values for added fats ranged from 0.59 (other starches) to 0.81 (vegetables). Comparisons between staff and participant scores for Five Food Group and Healthfulness scale scores resulted in correlations of 0.75 (p < .001) and 0.85 (p < .001), respectively.

## Discussion

This study describes the development and validation of an instrument to assess the types of foods served at home for the evening meal. The screener was developed to include a full range of foods that may be served at meals, particularly the evening meal, and a variety of healthful and unhealthful preparation methods. Study findings indicate the screening instrument has substantial criterion validity, and the checklist-type format was easily completed by participants in their homes.

The new screening instrument demonstrated criterion validity with moderate to high kappa values between participants' and staffs' reports of foods served at meals in the home and significant correlations between their scale scores regarding foods from the five major food groups and the healthfulness of foods. These findings and the fact that participants easily completed the screener suggests this tool can be used to effectively assess the types of foods served at meals. Costs and time associated with data collection in research studies could be reduced since participants are able to complete the screener in their own homes without research staff.

The Five Food Group and Healthfulness scales and most of the food categories showed substantial criterion validity; however, two comparisons resulted in kappas of less than 0.60. The general question of whether or not other starches were served had only moderate criterion validity. A detailed examination of these data indicates that staff were more likely to code "other starch" as present compared to participants. Perhaps the term "starch" is less commonly known among the general public even though pasta, noodles, potatoes, rice, and pizza dough were listed as examples. More research is needed in this area to assess how best to describe starchy carbohydrates on surveys. The suboptimal agreement between staff and participants regarding preparation method for vegetables resulted from the greater likelihood of staff reports of frying vegetables compared to participant reports. It may be that participants only recognize frying in deep fat as "frying." Future versions of the screener may separate out frying from sautéing to help increase validity.

The high average validity indices for added fats for vegetables and bread suggest that the screening instrument may be useful for studies interested in reducing butter and sauces as a form of weight control or to reduce cholesterol. In addition, the ease of completion with regard to time (participants completed the form as they prepared the meal) and convenience and the low cost of the data collection are great assets of this tool for population-based studies, particularly those promoting healthful foods such as salads, fresh vegetables, fruit for dessert, and milk consumption. Furthermore, although the screener was developed to assess the evening meal, further testing should be completed to evaluate its use for breakfast or lunch meals made at home.

To interpret the findings of this study, several issues warrant discussion. Study participants were self-selected volunteers and may not represent the general population in terms of motivation to complete the instrument and the types of meals prepared. In fact, some adults participated in several validation studies conducted by the research team, perhaps indicating a highly motivated group that may have been more conscientious in completing the screener, although none of our data or anecdotal evidence support this bias. Although the authors carefully considered many food varieties and those from different cultures, the screener may not capture all foods served at home meals and all methods of preparation (e.g., microwave cooking of protein) used by some families. Mixed dishes that contained many ingredients (e.g., soups) were more difficult to code on the instrument; however, problems were lessened when specific instructions were added during screener development. The instrument also includes additional "other" spaces for coding that could be used for foods common to a particular population. In addition, the screener does not assess the *quantity *of foods served at meals since participants either check "yes" or "no." However, our measure of a wide variety of different types of foods served at meals is similar to the variety score of the Healthy Eating Index [24] and may be linked to better diet quality. Our aim was to create a brief screener and keep response burden to a minimum. Attempting to collect data on more foods, quantities of foods, or more specifics about foods such as brand names would have compromised our aim. Lastly, the screener does not measure what was eaten at other eating occasions or at meals, only what was served at mealtime. In addition, the screener was designed to assess foods that are prepared in the home, limiting its utility for meals that are purchased elsewhere (i.e., takeout) but eaten in the home. Future research is needed to address the present study's limitations.

## Conclusions

To our knowledge, this meal screener is the first tool in the literature to assess the types of foods served at home meals and it proved to be a valid and participant-friendly tool that may be useful for research studies aiming to understand the home food environment, particularly those that are community-based where data collection is expensive and time-consuming. The screener adds a new and important meal component to the limited number of validated instruments that assess the home food environment. Furthermore, identifying the types and quality of foods served at home meals can help inform appropriate intervention strategies for individual households or might identify targets for public health messages. Future research should include more specific indices of healthfulness and assess the instrument's construct validity and test-retest reliability.

## Competing interests

The authors declare that they have no competing interests.

## Authors' contributions

JF conceptualized the study design, drafted the original screening tool, field tested the tool, requested input from experts in the field, trained staff for data collection, conducted the data analysis, and drafted the manuscript. LL and MS assisted with conceptualizing the study design and developing the screening tool as well as revising the manuscript for critical intellectual content. SM coordinated all data collection, data cleaning and data entry, contributed to protocol/instrument revisions and revised the manuscript for critical intellectual content. AS collected data and contributed to protocol/instrument revisions. AW contributed to instrument revisions and concept scoring. All authors have given final approval of the final version of the manuscript.
